# Temperature Resistance Properties of Unidirectional Laminated C*_f_*/SiC-Al Prepared by PIP and Vacuum Pressure Infiltration

**DOI:** 10.3390/ma16155445

**Published:** 2023-08-03

**Authors:** Tianru Guan, Le Lu, Zhaofeng Chen, Lixia Yang

**Affiliations:** International Laboratory for Insulation and Energy Efficiency Materials, College of Materials Science and Technology, Nanjing University of Aeronautics and Astronautics, Nanjing 211106, China; guantianru@nuaa.edu.cn (T.G.); lule0312@nuaa.edu.cn (L.L.)

**Keywords:** C*_f_*/SiC-Al, heat treatment, mechanical properties, microstructure

## Abstract

Material used for aero-engine fan blade requires excellent mechanical properties at high temperature (300 °C). Continuous carbon-fiber-reinforced silicon carbide ceramic matrix composites (C*_f_*/SiC) are necessary candidates in this field, possessing low density, high strength, high modulus, and excellent high-temperature resistance. However, during the preparation process of C*_f_*/SiC, there were inevitably residual pores and defects inside, resulting in insufficient compressive strength and reliability. The vacuum pressure melting infiltration process was used to infiltrate low melting point and high wettability aluminum alloys into the porous C*_f_*/SiC composite material prepared by the precursor impregnation cracking process, repairing the residual pore defects inside the body. The porosity of porous C*_f_*/SiC decreased from 49.65% to 5.1% after aluminum alloy repair and strengthening. The mechanical properties of C*_f_*/SiC-Al composite materials strengthened by aluminum alloy repair after heat treatment were studied. The tensile strength of the as-prepared C*_f_*/SiC-Al was 166 ± 10 MPa, which were degraded by 13~22% after heat treatment. The nonlinear sections of stress-displacement curve of as-treated samples were shorter than that of as-prepared sample. The hardness of aluminum alloy matrix after 300 °C 1 h heat treatment was 58 Hv, which was not obviously reduced compared with the sample without heat treatment. The vacuum infiltration of aluminum alloy is expected to have guiding significance for repairing and strengthening internal defects in ceramic matrix composites.

## 1. Introduction

Due to high specific strength, low density, and excellent high-temperature resistance, continuous fiber-reinforced ceramic matrix composites (CMC) have become the most candidate materials for medium to high-temperature applications (300~600 °C) [1,2]. Continuous fiber-reinforced aluminum matrix composites have been widely used in the aerospace field because of their excellent performance stability and good thermal conductivity [3,4]. However, aluminum alloy matrix is not competent for temperature-resistant structural load-bearing parts above 300 °C. Compared with continuous fiber-reinforced metal matrix composites, CMC has the advantages of lower density, higher stiffness, and high temperature resistance. Due to the limitations of the preparation process, CMC inevitably remains about 5~15% volume fraction of pore defects and has significant nonlinear behavior due to matrix cracking and interface debonding during heat treatment [5]. Callaway et al. analyzed its mechanical properties [6], which was mainly determined by fibers and interface layers. The interface debonding stress and failure mechanism of C/SiC were studied using Acoustic emission technology by Li et al. [7]. The monotonic tensile, cyclic loading and unloading behaviors of 3D-needle-punched C/SiC were studied by Liu et al. [8]. Heat treatment, as a technology for modifying many kinds of metals, has been widely used in industrial production. The experimental results showed that different temperatures have great influence on the mechanical strength of C/SiC and SiC/Si matrix composites. The effect of heat treatment on mechanical properties of C/SiC and SiC/SiC was studied by works [9,10,11,12,13]. The high temperature mechanical properties of CMC were researched by works [14,15,16,17,18]. Thermal residual stress is known to have a significant influence on macroscopic mechanical behavior of C/SiC while affecting the damage evolution. Therefore, the aluminum alloy infiltrated into the porous CMC without repair and strengthening can not only significantly reduce the porosity of CMC but also use the melting point of aluminum alloy during heat treatment to repair the matrix cracking and interface debonding of CMC.

Aluminum alloy was introduced into the porous C/SiC to prepare dense C/SiC-Al by combining precursor infiltration and pyrolysis (PIP), chemical vapor infiltration (CVI), and the vacuum pressure infiltration process; furthermore, the compressive properties and failure mechanism were studied by Liao and Xue [19,20]. Chopped fibers and silicon carbide particles were dispersed into aluminum particles by ball milling method, and then the mixture was hot pressed to prepared composites by Sha et al. [21]. The result showed that the tensile strength and Vickers hardness increased by 71% and 88%, respectively. Using aluminum alloy as raw material, aluminum alloy matrix was prepared by extrusion casting and spark plasma technology [22,23]. Through experiment and theoretical analysis, the mechanical properties of carbon fiber and aluminum matrix composites at different temperatures, and the mechanical properties of carbon fiber and aluminum matrix composites at different temperatures, were discussed [24]. The influence of the thickness of Al_2_O_3_ interface on mechanical properties of carbon-fiber-reinforced aluminum matrix composites was studied by Zhu et al. [25], which showed that 100 nm was the optimal thickness value. C*_f_*/Al were prepared by semi-solid rolling, and the infiltration behavior, mechanism, and mechanical properties were analyzed in-depth by Zhang et al. [26].

In this paper, continuous carbon-fiber-reinforced silicon carbide composites (C*_f_*/SiC) were prepared by PIP process, and the C*_f_*/SiC composites were repaired and strengthened by vacuum pressure impregnation. Unidirectional-laminated carbon fiber preforms were used as the skeleton of silicon carbide matrix and the main contributor to the mechanical properties of composites. The temperature resistance of the aluminum matrix composites was improved significantly because of the excellent stability at high temperature of silicon carbide matrix. The porosity was significantly reduced by aluminum alloy matrix and the mechanical properties were improved. To promote the application of repair and strengthening C*_f_*/SiC-Al composites in aero-engine fan blades (withstand temperature of 300 °C), the C*_f_*/SiC-Al composite’s temperature resistance properties were studied, and the mechanism was analyzed.

## 2. Experimental Procedure

T700 12 K carbon fiber was obtained from Toray Inc., Tokyo, Japan. Polycarbosilane (PCS) was purchased from Fujian Liya Chemical Co., Ltd., Fujian, China. Al alloy (ZL101A Si: 6.5–7.5; Mg: 0.25–0.45; Ti: 0.8; Al: remainder) was provided by Tianjin Boyat Metal Materials Trading Co., Ltd., Tianjin, China. All the other reagents were of analytical purity and were supplied by China National Pharmaceutical Group Chemical Reagent Co., Ltd., Shanghai, China. Propylene and argon (Ar) were obtained through Nanjing Chuangda Special Gas Co., Ltd., Nanjing, China.

Unidirectional-laminated carbon fiber preforms were used as reinforced skeleton with a volume fraction of 40%. C*_f_*/SiC-Al were fabricated by PIP and vacuum pressure infiltration processes. Firstly, pyrolytic carbon (PyC) with a thickness of ~300 nm was prepared on carbon fiber fabric by CVI at 1000 °C and 500 Pa, propylene was used as the source of carbon while Ar as the carrier gas. Secondly, carbon fiber preforms with PyC interphase were dipped into precursor solution that PCS took up 50 wt% for 2 h and dried at 150 °C for 6 h, then pyrolyzed under Ar atmosphere at 1200 °C for 1 h. The porous C*_f_*/SiC (porosity ~49.65% ± 0.05) were prepared after one infiltration and pyrolysis process.

**Figure 1:**
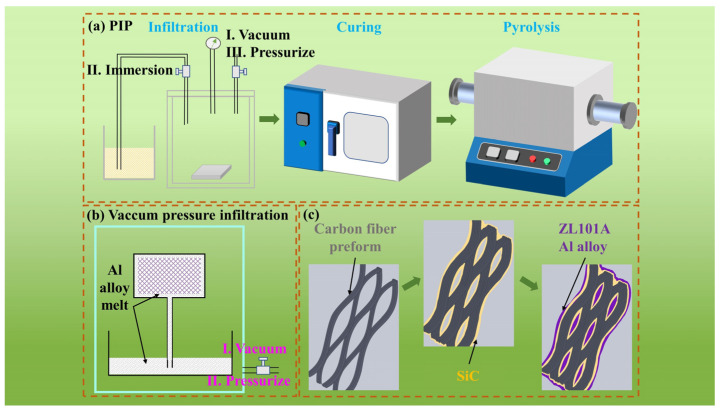
Schematic diagram of the preparing processes of C*_f_*/SiC-Al. (**a**) preparation of C*_f_*/SiC by PIP; (**b**) preparation of C*_f_*/SiC-Al by vacuum infiltration of aluminum alloy; (**c**) aluminum alloy repair and reinforcement of C*_f_*/SiC-Al.

The macroscopic image, pore structure, and pore size distribution of porous C*_f_*/SiC are shown in Figure S1 in the Supplementary Literature. The porous C*_f_*/SiC were prepared after one infiltration–pyrolysis process. Finally, vacuum pressure infiltration process was conducted at 750 °C and 1.2 MPa Ar pressure, Zl101A Al alloy was introduced into porous C*_f_*/SiC, then dense C*_f_*/SiC-Al were manufactured. The density and porosity of C*_f_*/SiC-Al have been studied in a previous work [27]. The detailed preparation process was given in Ref. [20]. Samples were heat-treated, respectively, at 200 °C for 1 h, 300 °C for 10 min, and 1 h in air (heated with the furnace at a heating rate of 5 °C/min and cooled in the air), then as-treated specimen were called S1, S2, and S3. At the same time, specimens without treatment called S0 were used to compare the effect of heat treatment on mechanical properties. Figure 1 shows the entire fabrication process.

Monotonic tensile tests of the as-prepared and as-treated samples were conducted at room temperature on a universal mechanical testing machine (CMT-5105, SHSAS, Nanjing, China), with a 100 KN load cell under displacement control and a loading rate of 5 mm/min. The geometry of the samples used for test was 90^L^ × 9.3 ± 1^W^ × 3.3 ± 0.2^T^ mm^3^. The surface and fracture morphology of the tested samples were characterized by a scanning electron microscope (SEM, Zeiss Supra 55, Oberkochen, German).

## 3. Results and Discussion

### 3.1. Mechanical Properties

**Figure 2:**
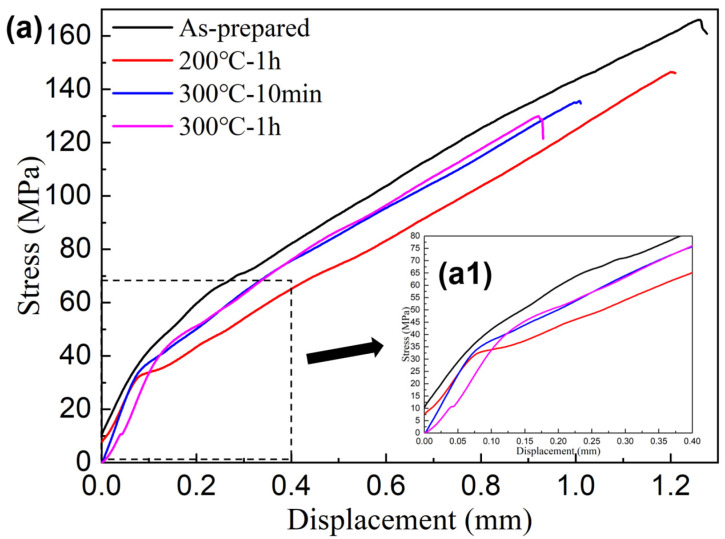
(**a**) Typical tensile stress-displacement curve of C*_f_*/SiC-Al; (**a1**) enlarged image of the box selection area.

Tensile properties of as-prepared and as-treated C*_f_*/SiC-Al were measured. Figure 2 showed the typical tensile stress-displacement curves of the as-prepared specimen (S0) and the as-treated specimen (S1: 200 °C-1 h; S2: 300 °C-10 min; S3: 300 °C-1 h). As shown in Figure 2a, the tensile stress-displacement curves of all specimen can be divided into three parts: initial linear section, nonlinear section, and second linear section. Figure 2(a1) presents an enlarged view of the partial area in Figure 2a, and the non-linear section of the as-treated samples became shortened significantly. The tensile strength of the as-prepared C*_f_*/SiC-Al tensile specimen was 166 ± 10 MPa, while that of the as-treated samples was reduced. Compared with S0, the tensile strengths of S1, S2, and S3 were degraded by 13%, 18%, and 22%, respectively. The elastic modulus changed little after heat treatment, which was calculated based on the slope of the initial linear segment. The failure displacement of as-treated samples decreased, and the work of fracture estimated by calculating the area under the stress-displacement curves gradually decreased, indicating the decreasing toughness [11].

The difference in the slope of first linear segment was small between as-treated and as-prepared samples. Heat treatment had little influence on the elastic modulus of C*_f_*/SiC-Al. The elastic modulus of C*_f_*/SiC-Al can be estimated by the mixing ratio criterion [9]:

(1) $$E=\lambda V_{f}E_{f}+V_{\mathrm{SiC}}E_{\mathrm{SiC}}+V_{\mathrm{Al}}E_{\mathrm{Al}}$$

where E is the elastic modulus of C*_f_*/SiC-Al, *V* is the volume fraction of each component while *E* is modulus, λ is the fiber distribution coefficient of preform.

The axis of the unidirectional-laminated carbon fiber preforms was parallel to the loading direction, so the value of λ is 1. In fact, the heat treatment under 400 °C almost has no effect on the mechanical properties of carbon fiber and silicon carbide matrix. The volume fraction of aluminum alloy in C*_f_*/SiC-Al was ~40%. However, the elastic modulus of ZL101A Al alloy was about 70 GPa, much smaller than that of T700 carbon fiber (230 GPa). Combining Formula (1), the modulus of the initial linear segment of C*_f_*/SiC-Al was mainly determined by carbon fiber, so that the effect of heat treatment on the elastic modulus of C*_f_*/SiC-Al was not obvious.

After heat treatment, the reduction in matrix-cracking stress indicated that the cracking stress of the matrix became smaller, which was related to the release of part of the residual stress. At the same time, the saturated cracking pressure of the matrix also reduced, and the range of the nonlinear section was also significantly shortened [28]. The nonlinear section of tensile stress-displacement curve was caused by toughening mechanisms such as matrix cracking, interface debonding, and slippage. When tensile load was applied, the non-linear section of the as-treated specimen became significantly shorter. The thermal expansion coefficients of carbon fiber, silicon carbide matrix, and aluminum alloy matrix were quite different. Residual stress caused by thermal mismatch in the process of cooling from preparation temperature to room temperature [14]. Because it has a higher coefficient of thermal expansion than carbon fiber and SiC, it has a certain pressure effect in cooling. The expression of residual stress in matrix is [29]:

(2) $$\sigma_{\mathrm{trs}}=E_{m}\frac{\lambda E_{f}V_{f}}{\lambda E_{f}V_{f}+E_{m}V_{m}}(\alpha_{f}-\alpha_{m})(T_{0}-T_{p})$$

where $\sigma_{\mathrm{trs}}$ is the thermal residual stress, α is the thermal expansion coefficient, $T_{0}$ is the operation temperature, $T_{p}$ is the processing temperature.

Compared with carbon fiber and aluminum alloy matrix, the failure strain of silicon carbide matrix is more minor. Both the shortening of the non-linear section and the reduction in the matrix-cracking saturation stress was caused by the release of residual thermal stress. Matrix cracking and interface debonding had an essential influence on the strength of C*_f_*/SiC-Al. When the as-treated sample was loaded, the matrix cracked and promoted the destruction of composites under a lower stress level.

The slope of the second linear segment in Figure 2 was almost the same, which indicated that the modulus of the second linear part did not change much. The second linear segment of C*_f_*/SiC-Al appeared because the load was entirely borne by the fiber actually. Therefore, the modulus of the second linear segment of the stress-displacement curve has little difference. The following formula can estimate modulus of C*_f_*/SiC-Al:

(3) $$E^{\prime }=\lambda V_{f}E_{f}$$

where $E^{\prime }$ is the modulus of the second linear segment.

### 3.2. Microstructure Analysis of C_f_/SiC-Al

**Figure 3:**
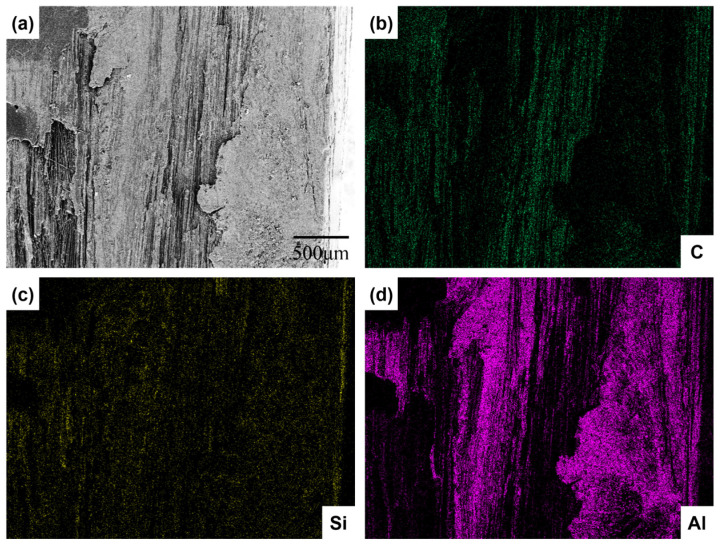
(**a**) SEM image of C*_f_*/SiC-Al after 1 h of heat treatment at 300 °C; (**b**–**d**) elemental mapping images of C*_f_*/SiC-Al after 1 h of heat treatment at 300 °C.

Figure 3 showed the cross-section SEM micrographs and element distribution of C*_f_*/SiC-Al. Only one PIP process was performed to prepare C*_f_*/SiC-Al in this work, so that the content of SiC matrix was less than that of Al alloy matrix, which was consistent with the element distribution. Although the carbon fiber was covered by the matrix, the distribution of the fiber can still be distinguished from the SEM photos. Dark fields appeared in the contact part between Al alloy matrix and carbon fiber, silicon carbide matrix as the sign of “stratified”, which indicated the low bonding strength.

**Figure 4:**
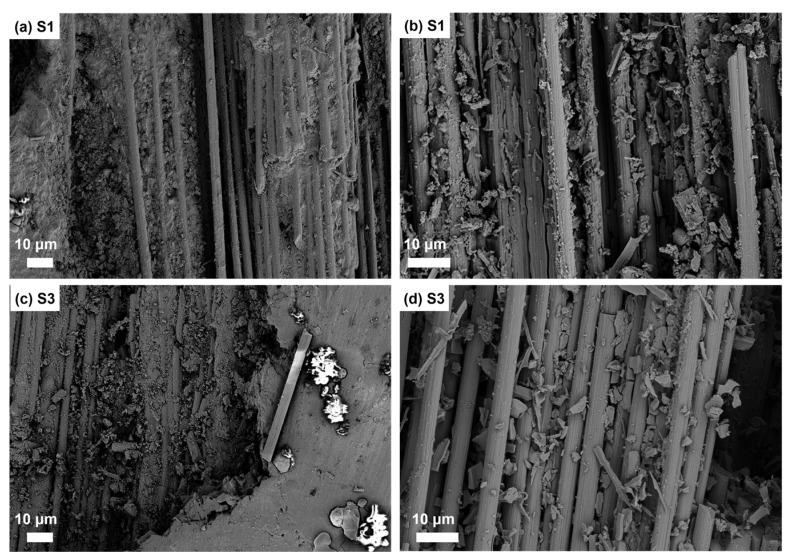
Fracture region micrographs of (**a**,**b**) C*_f_*/SiC-Al after 1 h of heat treatment at 200 °C; (**c**,**d**) C*_f_*/SiC-Al after 1 h of heat treatment at 300 °C.

Figure 4 showed fracture region micrographs of S1 and S3. It can be seen from Figure 4a that not only were the fiber filaments pulled out, multiple fibers with the matrix were pulled out as a whole, which showed a specific bonding strength between the fiber and SiC matrix. The cracks in the matrix can be seen in Figure 4c. The matrix was severely damaged and broken into small pieces as shown in Figure 4b,d. In addition, some substrates with poor wettability were in the state of “droplet”, which can be inferred to be Al alloy from their brightness. The matrix fracture, interfacial debonding, fiber fracture, and drawing failure existed in C*_f_*/SiC-Al matrix composites under tension, which makes the mechanical properties of the composites change nonlinearly. There was no significant difference in the failure mechanism after heat treatment.

### 3.3. Hardness Comparison before and after Heat-Treatment

**Figure 5:**
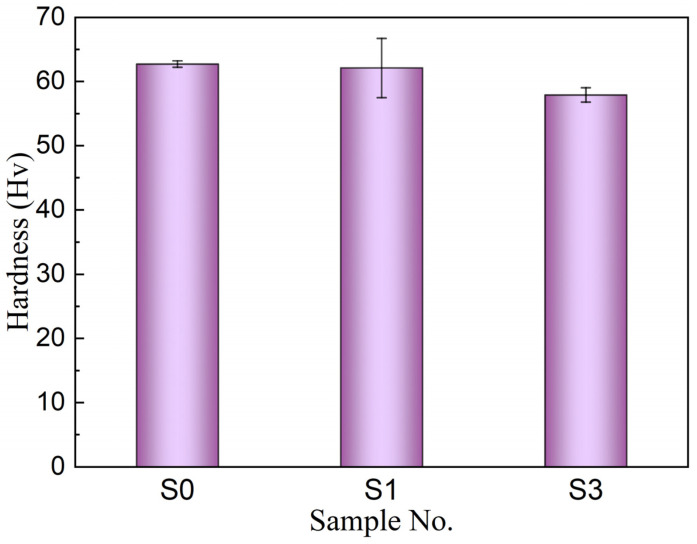
Hardness of aluminum alloy in C*_f_*/SiC-Al before and after heat treatment.

The Vickers hardness changes of the aluminum alloy matrix in C*_f_*/SiC-Al before and after heat treatment is shown in Figure 5. Among the various components of C*_f_*/SiC-Al, the performance of the aluminum alloy matrix was more easily affected at a high temperature of 300 °C. Currently, no characterization technology can separate the aluminum alloy matrix part in composite materials and study the changes in their tensile strength. Therefore, the effect of heat treatment on the mechanical properties of aluminum alloys in composite materials was characterized by Vickers hardness. The hardness of S1 sample was approximately 63 Hv, while the hardness of S2 sample was approximately 58 Hv. Compared to S1, the hardness of S2 decreased by approximately 7.9%. The dispersion of Vickers hardness of the aluminum alloy matrix in S1 sample was relatively large. After heat treatment, the hardness of the aluminum alloy tended to decrease, but the change was not significant. After heat treatment, the hardness and strength of ZL101A aluminum alloy decreased, and the overall mechanical properties of C*_f_*/SiC-Al composite material were also affected.

### 3.4. Mechanism Comparison before and after Heat-Treatment

**Figure 6:**
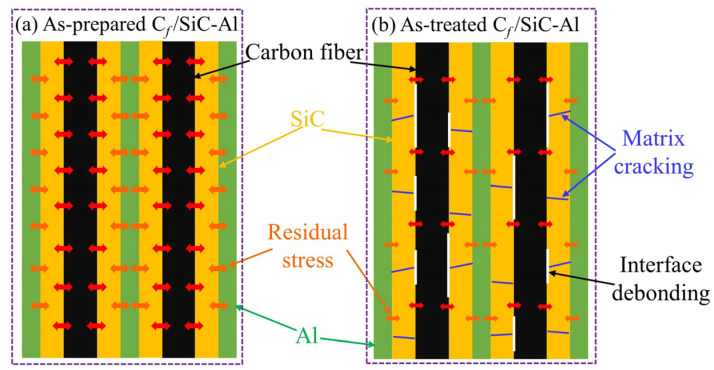
Schematic diagram of the influence mechanism on mechanical properties of heat treatment.

The tensile stress-displacement curves of untreated and treated composites had significant nonlinear sections. After heat treatment, the nonlinear area of the stress-displacement curves became shorter. The residual stress caused by the thermal mismatch of the components of C*_f_*/SiC-Al was released because of the introduction of heat energy, which resulted in matrix cracking and interface debonding, as shown in Figure 6. The matrix cracked under a lower load during the loading process, which was consistent with the reduction in the matrix-breaking stress and saturated matrix cracking stress of as-treated sample. When pressure reached the saturated matrix-cracking stress, the matrix cracks saturated, and the load was borne by fiber. Premature load-bearing and stress concentration will make the fiber more susceptible to damage, which was also the main reason for the decrease in tensile strength of as-treated sample. The hardness of the aluminum alloy in as-treated C*_f_*/SiC-Al did not degrade much and, so, it can be assumed that the mechanical properties of aluminum alloy after heat treatment did not change significantly. In addition, the modulus of aluminum alloy did not contribute much to the modulus of C*_f_*/SiC-Al; thus, the effect of heat treatment on aluminum alloy matrix did not have a major impact on the performance of C*_f_*/SiC-Al.

## 4. Conclusions

The strength of C*_f_*/SiC-Al after 300 °C-treatment reduced by 22%, which was related to the premature load-bearing and stress concentration of fiber caused by reduction in matrix-cracking stress and saturated matrix-cracking stress. The modulus of the first linear segment and the second linear segment of C*_f_*/SiC-Al after heat treatment had no obvious tendency to degrade. Carbon fiber and silicon carbide matrix were the main contributors to the modulus of C*_f_* /SiC-Al. The hardness of S1 sample was approximately 63 Hv, while the hardness of S2 sample was approximately 58 Hv. Compared to S1, the hardness of S2 decreased by approximately 7.9%. C*_f_*/SiC-Al matrix fracture, interfacial debonding, fiber fracture, and fiber detachment were the failure mechanisms of C*_f_*/SiC-Al matrix fracture and fiber detachment, while 200 and 300 °C had little effect on the failure mechanism of C*_f_*/SiC-Al matrix, respectively. Aluminum alloy infiltration repair can effectively reduce internal defects in materials and improve the reliability of composite materials. It is expected to realize the rapid densification of ceramic matrix composites, shorten the material preparation cycle, and reduce costs, with significant economic benefits.

## Data Availability

Exclude this statement.

## Supplementary Materials

The following supporting information can be downloaded at: https://www.mdpi.com/article/10.3390/ma16155445/s1, Figure S1: CT images of porous C*_f_*/SiC cross-section.
