# Supplementary material for: Temperature Resistance Properties of Unidirectional Laminated Cf/SiC-Al Prepared by PIP and Vacuum Pressure Infiltration
Source: Materials (Basel). 2023 Aug 3;16(15):5445. doi: 10.3390/ma16155445 (PMC10420298; doi:10.3390/ma16155445)
Supplement: Supplementary file 1 [file materials-16-05445-s001.zip › materials-2376518-supplementary.pdf]

materials

Supplementary Materials

Temperature Resistance Properties of Unidirectional Laminated C_f/SiC-Al Prepared by PIP and Vacuum Pressure Infiltration

Tianru Guan, Le Lu, Zhaofeng Chen * and Lixia Yang *

International Laboratory for Insulation and Energy Efficiency Materials, College of Materials Science and Technology, Nanjing University of Aeronautics and Astronautics, Nanjing, 211106, China; guantianru@nuaa.edu.cn (T.G.); lule0312@nuaa.edu.cn (L.L.)
* Correspondence: zhaofeng_chen@163.com (Z.C.); lixiayang@nuaa.edu.cn (L.Y.)

Citation: Guan, T.; Lu, L.; Chen, Z.; Yang, L. Temperature Resistance Properties of Unidirectional Laminated C_f/SiC-Al Prepared by PIP and Vacuum Pressure Infiltration. *Materials* **2023**, *16*, 5445. https://doi.org/10.3390/ma16155445

Received: 30 June 2023
Revised: 26 July 2023
Accepted: 27 July 2023
Published: 3 August 2023

Copyright: © 2023 by the authors. Submitted for possible open access publication under the terms and conditions of the Creative Commons Attribution (CC BY) license (https://creativecommons.org/licenses/by/4.0/).

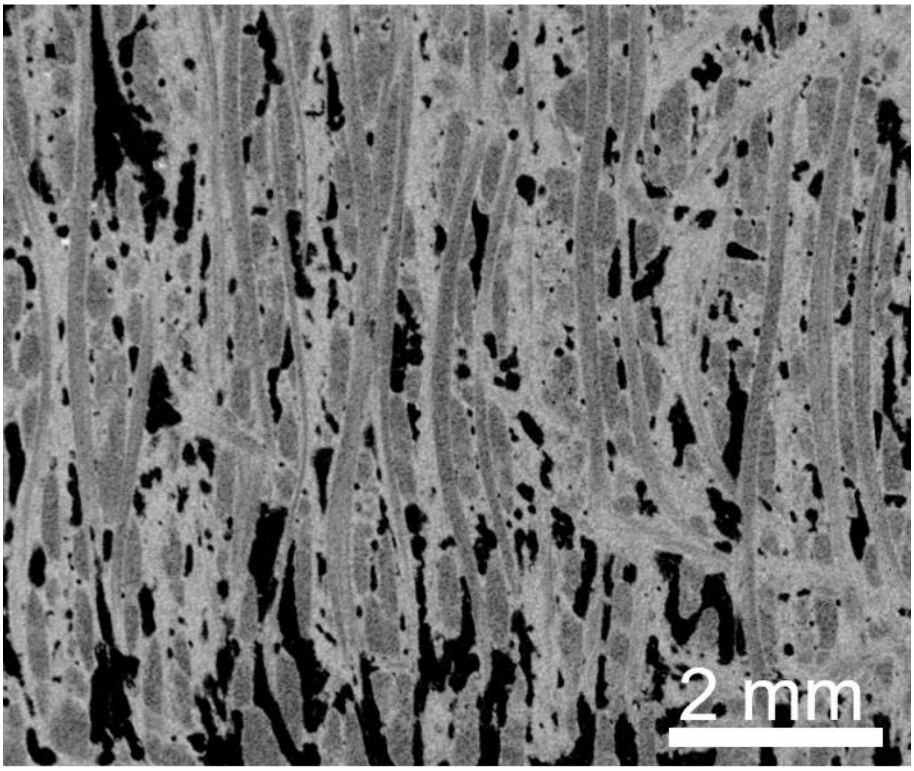

Figure S1. CT images of porous Cf/SiC cross-section.
